# SPECT imaging of distribution and retention of a brain-penetrating bispecific amyloid-β antibody in a mouse model of Alzheimer’s disease

**DOI:** 10.1186/s40035-020-00214-1

**Published:** 2020-09-21

**Authors:** Tobias Gustavsson, Stina Syvänen, Paul O’Callaghan, Dag Sehlin

**Affiliations:** 1 Department of Public Health and Caring Sciences, Uppsala University, Uppsala, Sweden; 2 Department of Medical Cell Biology, Uppsala University, Uppsala, Sweden

**Keywords:** Alzheimer’s disease, Amyloid beta, SPECT, Immunotherapy, Bispecific antibody, Transferrin receptor

## Abstract

**Background:**

Alzheimer’s disease (AD) immunotherapy with antibodies targeting amyloid-β (Aβ) has been extensively explored in clinical trials. The aim of this study was to study the long-term brain distribution of two radiolabeled monoclonal Aβ antibody variants – RmAb158, the recombinant murine version of BAN2401, which has recently demonstrated amyloid removal and reduced cognitive decline in AD patients, and the bispecific RmAb158-scFv8D3, which has been engineered for enhanced brain uptake via transferrin receptor-mediated transcytosis.

**Methods:**

A single intravenous injection of iodine-125 (^125^I)-labeled RmAb158-scFv8D3 or RmAb158 was administered to AD transgenic mice (tg-ArcSwe). In vivo single-photon emission computed tomography was used to investigate brain retention and intrabrain distribution of the antibodies over a period of 4 weeks. Activity in blood and brain tissue was measured ex vivo and autoradiography was performed in combination with Aβ and CD31 immunostaining to investigate the intrabrain distribution of the antibodies and their interactions with Aβ.

**Results:**

Despite faster blood clearance, [^125^I]RmAb158-scFv8D3 displayed higher brain exposure than [^125^I]RmAb158 throughout the study. The brain distribution of [^125^I]RmAb158-scFv8D3 was more uniform and coincided with parenchymal Aβ pathology, while [^125^I]RmAb158 displayed a more scattered distribution pattern and accumulated in central parts of the brain at later times. Ex vivo autoradiography indicated greater vascular escape and parenchymal Aβ interactions for [^125^I]RmAb158-scFv8D3, whereas [^125^I]RmAb158 displayed retention and Aβ interactions in lateral ventricles.

**Conclusions:**

The high brain uptake and uniform intrabrain distribution of RmAb158-scFv8D3 highlight the benefits of receptor-mediated transcytosis for antibody-based brain imaging. Moreover, it suggests that the alternative transport route of the bispecific antibody contributes to improved efficacy of brain-directed immunotherapy.

## Background

Currently, approximately 45 million people worldwide are affected by Alzheimer’s disease (AD) [1]. To date, there is no treatment that can halt the neurodegenerative processes of AD. Biopharmaceutical drugs, such as monoclonal antibodies (mAbs), have advantages of high affinity and target specificity combined with the ability to initiate multiple different downstream effector functions, facilitating treatment effects [2].

Intracellular neurofibrillary tangles of hyperphosphorylated tau and extracellular plaques composed of aggregated amyloid-β (Aβ) are pathological hallmarks of AD [3]. Although large insoluble Aβ fibrils are the main constituent of plaques, the smaller soluble oligomeric and protofibrillar Aβ species have been suggested to be linked to disease progression [4] and impaired synaptic function [5, 6], and may thus provide a better therapeutic target. Multiple AD-modifying immunotherapeutic approaches have been explored, but many have failed. BAN2401, a conformation-dependent Aβ mAb that binds selectively to soluble Aβ oligomers and protofibrils [7, 8], reduced Aβ load and slowed the cognitive decline in a dose-dependent manner in a large phase 2b study of 856 prodromal and mild AD patients [9]. BAN2401 was generally well-tolerated with low incidence (< 10% at the highest dose) of amyloid-related imaging abnormalities – edema (ARIA-E). The majority of the events were asymptomatic and typically resolved within 4–12 weeks [9]. ARIA-E is suggested to be initiated by interactions with Aβ deposited in the brain vasculature, i.e. cerebral amyloid angiopathy (CAA) [10], causing a dose-dependent BBB disruption due to inflammatory processes in the vasculature [11].

Brain entry of large and hydrophilic molecules is severely limited by the blood-brain barrier (BBB) and the blood-cerebrospinal fluid barrier (BCSFB). The BBB is composed of tightly connected endothelial cells, pericytes, and astrocytic endfeet projections that together form the neurovascular unit [12]. The BCSFB separates the cerebrospinal fluid and systemic circulation, and is formed by tight junction connections between epithelial cells at the choroid plexus [13]. The disadvantage of these barriers, from the neuropharmacological perspective, is a reduced brain uptake of large molecules such as antibodies, limiting their brain exposure [14]. Bispecific antibodies that display dual-target affinity, for example targeting an endogenous BBB transport mechanism and a brain parenchymal target, such as Aβ, have been developed to increase brain uptake [15]. One such BBB transporting system is the transferrin receptor (TfR), which transports the iron-binding protein transferrin across the endothelium of the BBB [16]. The TfR can also be used to deliver biological drugs across the BBB. The exact mechanism of TfR-mediated transcytosis is under debate, which has been further detailed in an extensive review by Johnsen et al. [17]. Multiple bispecific antibody constructs have been created that utilize TfR to improve brain parenchymal exposure to mAbs [18–22].

We have previously developed a bispecific mAb, RmAb158-scFv8D3, based on RmAb158 (recombinant murine version of BAN2401) that is fused to a single-chain variable fragment (scFv) of the mouse TfR mAb 8D3 [18]. RmAb158-scFv8D3 can rapidly cross the BBB and displays faster blood clearance than the unmodified RmAb158 [23]. We have demonstrated that the radiolabeled RmAb158-scFv8D3 can be used as a positron emission tomography (PET) ligand to visualize and quantify Aβ aggregates in vivo and to quantify the change in Aβ load following anti-Aβ treatment [24]. Furthermore, in an acute therapeutic application, RmAb158-scFv8D3 at a ten times lower dose is as potent as its unmodified form RmAb158 in lowering profibrillar Aβ [23]. It is unclear how the rapid blood clearance of RmAb158-scFv8D3 affects the therapeutic efficacy of this bispecific antibody in a long-term perspective. It may potentially reduce side effects due to the shorter blood half-life, but at the expense of treatment efficacy.

In this study, we set out to longitudinally assess the brain retention and distribution of RmAb158-scFv8D3 and RmAb158 using single-photon emission computed tomography (SPECT), during a four-week period in the tg-ArcSwe mouse model of AD. Ex vivo brain autoradiography and nuclear track emulsion autoradiography, combined with Aβ and CD31 immunostaining, were performed to visualize where in the brain the antibodies were retained.

## Methods

### Radiochemistry

RmAb158-scFv8D3 and RmAb158 were directly labeled with ^125^I (half-life 59.5 days) using the Chloramine-T method [25]. Briefly, 70 μg of antibody was radioiodinated with 60–70 MBq ^125^I stock solution (PerkinElmer Inc., Waltham, MA, USA) with the addition of 5 μg (200 μM) Chloramine-T (Sigma Aldrich, Stockholm, Sweden) and incubated for 90 s at room temperature. The reaction was quenched by addition of 10 μg (440 μM) of sodium metabisulfite (Sigma Aldrich). The final radiolabeling volume was 120 μl. The radiolabeled proteins were purified from free radioiodine using a NAP-5 size exclusion column with molecular-weight cut-off of 5 kDa (GE Healthcare AB, Uppsala, Sweden), and eluted in 700 μl PBS (pH 7.4).

### Binding kinetics

LigandTracer (Ridgeview Instruments AB, Vänge, Sweden) is a technique for real-time measurement of kinetic properties of protein-protein interaction on living cells or fixated proteins [26]. Polystyrene plates (Corning Inc., Corning, NY, USA) were coated with 1 μM synthetic Aβ42 protofibrils (American Peptide, Sunnyvale, CA, USA) prepared as previously described [19], and 0.2 μM mouse TfR (mTfR; in-house produced), and incubated at 4 °C for 24 h. The plates were blocked for 2 h at room temperature with 1% BSA in PBS, and the radiolabeled antibodies used in the assay were diluted in 0.1% BSA in PBS. Binding of [^125^I]RmAb158-scFv8D3 and [^125^I]RmAb158 to Aβ protofibrils was evaluated with LigandTracer using 0.3 nM and 1 nM antibody concentrations, and the binding to mTfR was evaluated using 1 nM and 3 nM antibody concentrations. Experiments were performed in triplicate. The antibody-target interactions were analyzed in TraceDrawer v.1.8.1 (Ridgeview Instruments AB) using a 1:1 kinetic fitting model with or without depletion correction.

### Animals

Eighteen- to 24-month-old wild-type (WT; *n* = 2, male) and tg-ArcSwe mice (*n* = 28/34, female/male) expressing human Aβ protein precursor (AβPP) with the Swedish (KM670/671NL) and the Arctic (E693G) AβPP mutations, were used in this study. All procedures described in this study were approved by the Uppsala County Animal Ethics board (C17/14 and 5.8.18–13,350/2017), and were in accord with the rules and regulations of the Swedish Animal Welfare Agency and complied with the European Communities Council Directive of 22 September 2010 (2010/63/EU).

### SPECT

Mice that underwent SPECT scanning were given water supplemented with 0.2% NaI throughout the study to reduce thyroidal uptake of free ^125^I. Mice were intravenously (i.v.) injected with 10.39 ± 1.91 MBq [^125^I]RmAb158-scFv8D3 (molar activity at the time of administration: 129 ± 32 MBq/nmol; dose: 0.65 ± 0.14 mg/kg) (*n* = 9) or 8.67 ± 0.84 MBq [^125^I]RmAb158 (molar activity at the time of administration: 117 ± 62 MBq/nmol; dose: 0.25 ± 0.07 mg/kg) (*n* = 5). SPECT scans were obtained at 3, 6, 14, and 27 days after injection. Each mouse underwent a maximum of three SPECT scans. Mice were anesthetized with 3% sevoflurane before scanning, and then positioned on the pre-heated scanner bed of the small animal nanoScan SPECT/CT (Mediso Medical Imaging Systems, Hungary). CT was performed with the following settings: 50 kilovoltage peak X-ray, 600 μA, and 480 projections; and CT images were reconstructed using filtered back projections. ^125^I γ emission was collected with an acquisition frame of 2 min.

SPECT acquisition data were reconstructed using Nuclide 2.03 software and Tera-Tomo^tm^ 3D SPECT reconstructive algorithm (Mediso Medical Imaging Systems, Hungary) with scattering and attenuation correction. SPECT images were reconstructed using 48 iterations into a static image. Reconstructed data were decay-corrected and adjusted for injected dose. Data were visualized in AMIDE v 1.0.4 (http://amide.sourceforge.net/).

### Ex vivo autoradiography

Ex vivo autoradiography was performed to visualize antibody retention in brain tissue. Coronal brain cryosections (50 μm) of SPECT-scanned mice and a radioactive standard were exposed to phosphor imaging plates (MS, MultiSensitive, PerkinElmer, Downers Grove, IL, USA) for 4 days. The plates were then scanned in a Cyclone Plus phosphor imager (PerkinElmer) at 600 dpi resolution. Radioactive distribution in brain sections was visualized with ImageJ using a royal lookup table. The images obtained were decay-corrected and normalized to the injected dose.

### Ex vivo and pharmacokinetic studies

Mice used only for ex vivo experiments (*n* = 42) were i.v. administered with 1.39 ± 1.03 MBq [^125^I]RmAb158-scFv8D3 (dose: 0.14 ± 0.11 mg/kg) or 2.09 ± 2.59 MBq [^125^I]RmAb158 (dose: 0.22 ± 0.11 mg/kg). Animals designated for nuclear track emulsion (NTE) autoradiography were injected with 1.70 ± 0.70 MBq [^125^I]RmAb158-scFv8D3 (*n* = 3) or 1.66 ± 0.97 MBq [^125^I]RmAb158 (*n* = 3).

Blood samples (8 μl) were taken from the tail vein at 4 h, 24 h, 48 h, 72 h, and 168 h post-injection (p.i.) and a terminal blood sample was obtained from the heart before mice were euthanized by intracardiac perfusion with 50 ml of 0.9% saline over 2 min. Brains were divided into a cerebral and a cerebellar part and frozen.

The activity in blood and brain samples was measured with a γ counter (1480 Wizard™, Wallac Oy, Turku, Finland). Antibody concentrations in cerebrum, cerebellum and blood were quantified as the percent of injected dose per gram tissue (%ID/g). Antibody exposure was quantified by area under curve (AUC) calculations, using the Prism 6 software (GraphPad Software, Inc., La Jolla, CA, USA).

### Aβ40 immunohistochemistry

Coronal cryosections of 50 μm were fixed in 4% formaldehyde for 20 min and washed in PBS, followed by antigen retrieval with 25 mM citric acid buffer (pH 7.3) and then 70% formic acid. Endogenous peroxidase was blocked with 0.3% hydrogen peroxide for 15 min, and then the sections were permeabilized with 0.4% Triton X-100 in PBS for 5 min, incubated overnight at 4 °C with a polyclonal anti-Aβ40 antibody (Agrisera, Umeå, Sweden, custom-made; 0.5 μg/ml in 0.1% Tween-20 in PBS), followed by 30-min incubation with biotinylated goat anti-rabbit IgG (Vector Laboratories Inc., Burlingame, CA) and 30-min incubation with Streptavidin-HRP (Mabtech AB). The sections were processed for colour development with Nova Red chromogen (Vector Laboratories Inc.) and imaged with a Zeiss Observer Z.1 microscope using ZEN 2.6 software (Carl Zeiss Microimaging GmbH, Jena, Germany).

### Immunofluorescence staining and NTE autoradiography

Cryosections of 20 μm were fixed in cold methanol for 10 min and blocked for 1 h with normal goat serum, followed by tissue permeabilization in 0.1% Tween 20 in PBS for 5 min. Then the sections were incubated overnight with rat anti-mouse CD31 (BD Biosciences, Catalog No. 553370; 1.25 μg/ml) or polyclonal rabbit anti-Aβ40 antibody (Agrisera; 0.5 μg/ml) at 4 °C, and incubated with Alexa-594-conjugated goat anti-mouse IgG or Alexa-488 goat anti-rat IgG for 1 h at room temperature.

NTE was performed in darkness according to the previously described protocol [19]. Following immunostaining, the sections were directly submerged in ILFORD K5 emulsion, air-dried for 2 h at room temperature and exposed for 2 weeks at 4 °C. The emulsion-covered tissue sections were developed according to the manufacturer’s instructions, dehydrated in an increased series of ethanol solution and mounted with Pertex mounting medium. The immunofluorescence and NTE stainings were imaged with a Zeiss Observer Z.1 microscope using the ZEN 2.6 software (Carl Zeiss Microimaging GmbH, Jena, Germany).

### Image processing and quantification

For visual representation, the NTE images were color-inverted and image information in histogram at tonal range of 20,000–43,000 was extracted to construct the NTE images. Rolling ball background subtraction was performed on immunofluorescence images.

To determine the degree to which the NTE signal was associated with Aβ40-positive deposits, the images of Aβ40-immunostained and NTE-developed sections from brain hemispheres of RmAb158- and RmAb158-scFv8D3-treated mice were opened in the Fiji platform [27] and processed using the ‘Subtract Background’ tool. A signal threshold for Aβ40-positive structure was applied to all sections and regions of interest (ROIs) were identified using the ‘Analyse Particles’ tool. These ROIs were superimposed on the corresponding NTE image and the mean NTE intensity was measured for each of the Aβ40-positive ROIs. To differentiate between NTE signals derived from the parenchyma and the vasculature, the images of CD31-immunostained and NTE-developed sections of RmAb158 or RmAb158-scFv8D3 treated mice were processed with the ‘Subtract Background’ tool. A signal threshold for CD31 immunosignal was applied and a binary mask was created. This identified the CD31-positive regions of the section. Using the ‘Image Calculator’ tool, the CD31-positive masked regions were subtracted from images of the corresponding NTE signal. This produced an image of NTE signal in CD31-negative (i.e. parenchymal) regions. A threshold was applied to these images of parenchymal NTE signal, and the ‘Particle Analysis’ tool was used to identify and count parenchymal NTE ROIs and to measure the total area of parenchymal NTE signal.

### Statistical analysis

Statistical analysis was performed using the Prism 6 software (GraphPad Software, Inc., La Jolla, CA, USA). Comparisons of AUC_blood_ and AUC_brain_ between [^125^I]RmAb158-scFv8D3- and [^125^I]RmAb158-treated animals were made using unpaired Welch’s *t*-test (non-parametric). The AUC_blood_ and AUC_brain_ data are presented as mean ± SEM. Otherwise, values are reported as mean ± SD. *P* < 0.05 was considered statistically significant.

## Results

### Real-time tracer binding

**Fig. 1:**
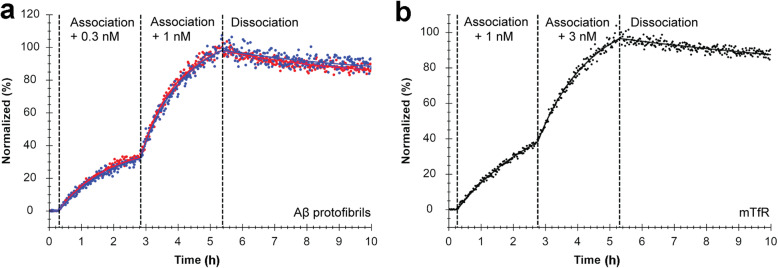
Representative normalized LigandTracer sensograms of (**a**) [^125^I]RmAb158-scFv8D3 (blue) and [^125^I]RmAb158 (red) binding to Aβ protofibrils, and of (**b**) [^125^I]RmAb158-scFv8D3 binding to mTfR. The dots represent raw data and solid lines represent curve fit using a 1:1 model with depletion correction

The binding characteristics of radiolabeled RmAb158-scFv8D3 and RmAb158 to Aβ protofibrils and TfR were evaluated with LigandTracer – a technique that enables real-time measurement of ligand-receptor kinetics [26]. [^125^I]RmAb158-scFv8D3 and [^125^I]RmAb158 displayed similar Aβ protofibril-binding, evaluated with a 1:1 fitting model with and without depletion correction (Table 1), the former (Fig. 1) assuming that a certain amount of ligand is depleted from the reaction solution during the association phase, thus yielding a better curve fit. However, both models resulted in similar values for association and dissociation rate constants (Table 1). With depletion correction, the binding affinities (*K*_D_) of [^125^I]RmAb158-scFv8D3 and [^125^I]RmAb158 to Aβ protofibrils (Fig. 1a) were estimated to be 80.7 ± 2.94 pM and 75.1 ± 3.22 pM, respectively. RmAb158-scFv8D3 had a lower affinity to mTfR (*K*_D_ 155 ± 30.6 pM) than to Aβ protofibrils (Fig. 1b, Table 1). The non-mTfR binding antibody, RmAb158, did not interact with mTfR (data not shown).

**Table 1:** [^125^I]RmAb158-scFv8D3 and [^125^I]RmAb158 binding properties

Ligand	Target	Model	*k*_a_ (M^−1^ s^− 1^)	*k*_d_ (s^− 1^)	*K*_D_ (pM)
[^125^I]RmAb158-scFv8D3 (*n* = 3)	Aβ protofibril	1:1 depletion corrected	1.96 × 10^5^ ± 2.74 × 10^4^	1.53 × 10^− 5^ ± 3.35 × 10^− 6^	80.7 ± 2.94
[^125^I]RmAb158 (*n* = 3)	Aβ protofibril	1:1 depletion corrected	1.84 × 10^5^ ± 1.56 × 10^4^	1.41 × 10^− 5^ ± 7.11 × 10^− 6^	75.1 ± 3.22
[^125^I]RmAb158-scFv8D3 (*n* = 3)	mTfR	1:1 depletion corrected	4.62 × 10^4^ ± 8.16 × 10^3^	7.13 × 10^− 6^ ± 1.67 × 10^− 6^	155 ± 30.6
[^125^I]RmAb158-scFv8D3 (*n* = 3)	Aβ protofibril	1:1	1.49 × 10^5^ ± 2.76 × 10^4^	1.12 × 10^− 5^ ± 1.39 × 10^− 6^	75.8 ± 6.05
[^125^I]RmAb158 (*n* = 3)	Aβ protofibril	1:1	1.40 × 10^5^ ± 1.01 × 10^4^	1.01 × 10^− 5^ ± 2.93 × 10^− 7^	71.9 ± 3.51
[^125^I]RmAb158-scFv8D3 (*n* = 3)	mTfR	1:1	3.91 × 10^4^ ± 5.76 × 10^3^	6.64 × 10^−6^ ± 2.16 × 10^− 6^	167 ± 37.6

Kinetic parameters of LigandTracer data from [^125^I]RmAb158-scFv8D3 and [^125^I]RmAb158 interacting with Aβ protofibrils and mTfR, using a 1:1 model with and without depletion correction. Values are mean ± SD

### SPECT imaging

**Fig. 2:**
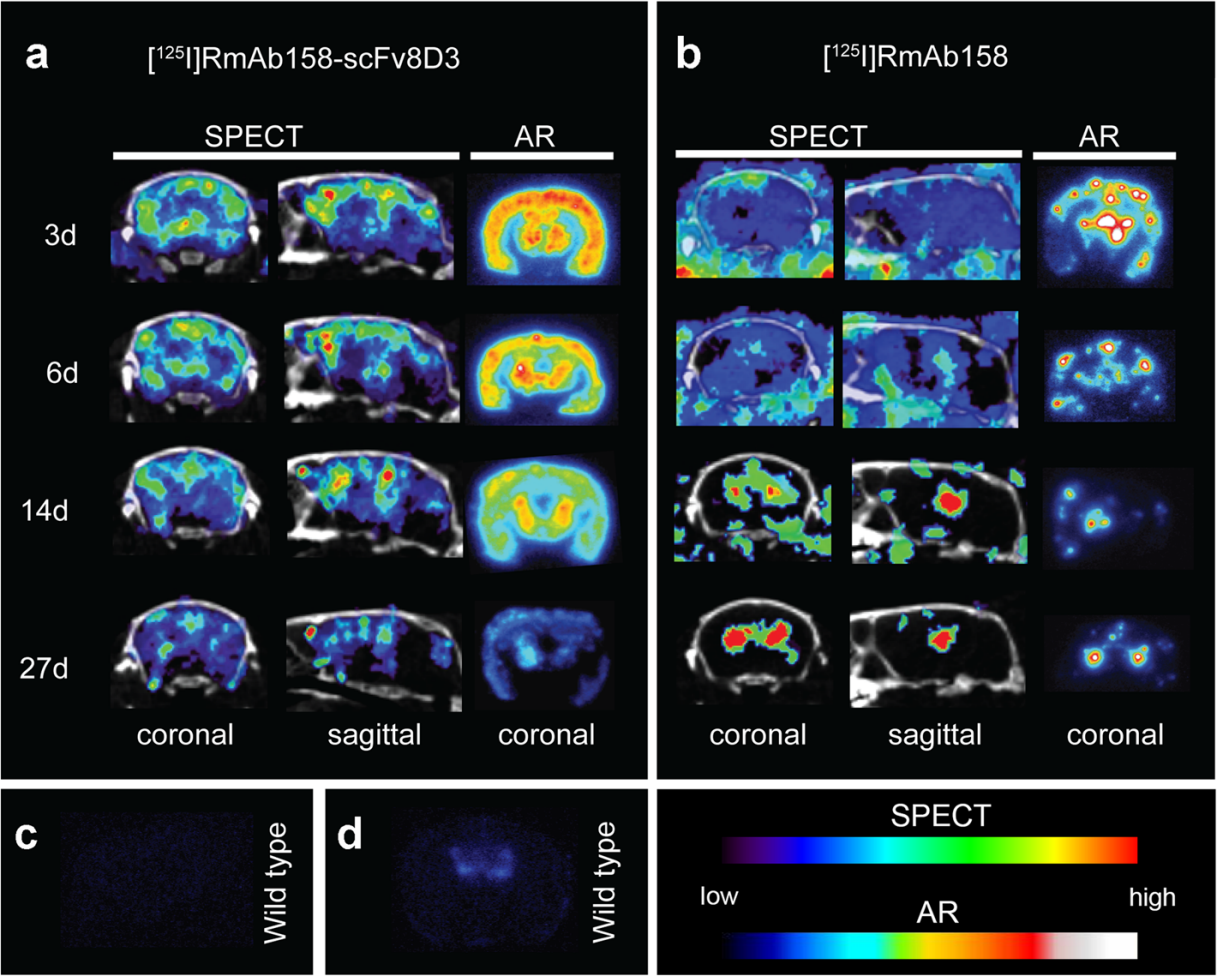
Coronal and sagittal SPECT images and coronal ex vivo autoradiography (AR) images of representative tg-ArcSwe mice from day 3 to day 27 after injection of [^125^I]RmAb158-scFv8D3 (**a**) (3 days: *n* = 4; 6 days: *n* = 4; 14 days: *n* = 3; 27 days: *n* = 5) or [^125^I]RmAb158 (**b**) (3 days: *n* = 2; 6 days: *n* = 3; 14 days: *n* = 4; 27 days: *n* = 3). **c**, **d** AR of WT mice injected with [^125^I]RmAb158-scFv8D3 (*n* = 1) (**c**) and [^125^I]RmAb158 (*n* = 1) (**d**) on day 3 post injection. Images were decay-corrected and normalized to injected dose

The tg-ArcSwe mice injected with [^125^I]RmAb158-scFv8D3 or [^125^I]RmAb158 underwent SPECT scanning on days 3, 6, 14, and 27 post-injection. [^125^I]RmAb158-scFv8D3 displayed high brain uptake and a uniform distribution pattern in areas with known Aβ pathology. The SPECT images showed that [^125^I]RmAb158-scFv8D3 resided in the brain at higher concentrations than [^125^I]RmAb158 during the whole study period of 27 days. In addition, the intrabrain distribution of [^125^I]RmAb158-scFv8D3 was spatially relatively stable during the study, with signals slowly decreasing in all brain regions over time. These observations were further confirmed with ex vivo autoradiography, which demonstrated a uniform distribution pattern and decreased antibody retention over time (Fig. 2a).

In contrast, [^125^I]RmAb158 displayed a fundamentally different distribution pattern (Fig. 2b). At 3 days post-injection, SPECT scanning revealed no detectable [^125^I]RmAb158 signal in Aβ-abundant brain areas, as the measured signals were mostly blood-derived background activity. At 6 days, [^125^I]RmAb158 retention was visible in central brain areas in two out of three scanned mice. At 14 and 27 days post-injection, SPECT scanning showed further increased [^125^I]RmAb158 retention in regions that appeared to be ventricles, in both the coronal and sagittal views (Fig. 2b). In contrast to [^125^I]RmAb158-scFv8D3, ex vivo autoradiography revealed hotspots of antibody retention in the cortex and central brain (Fig. 2b). There was a gradual loss of antibody from the cortex, while the central brain areas displayed a more sustained antibody signal.

[^125^I]RmAb158-scFv8D3 displayed no brain retention in WT mice 3 days post injection (Fig. 2c), while [^125^I]RmAb158 showed a faint signal centrally in the brain (Fig. 2d).

### Ex vivo tissue activity

**Fig. 3:**
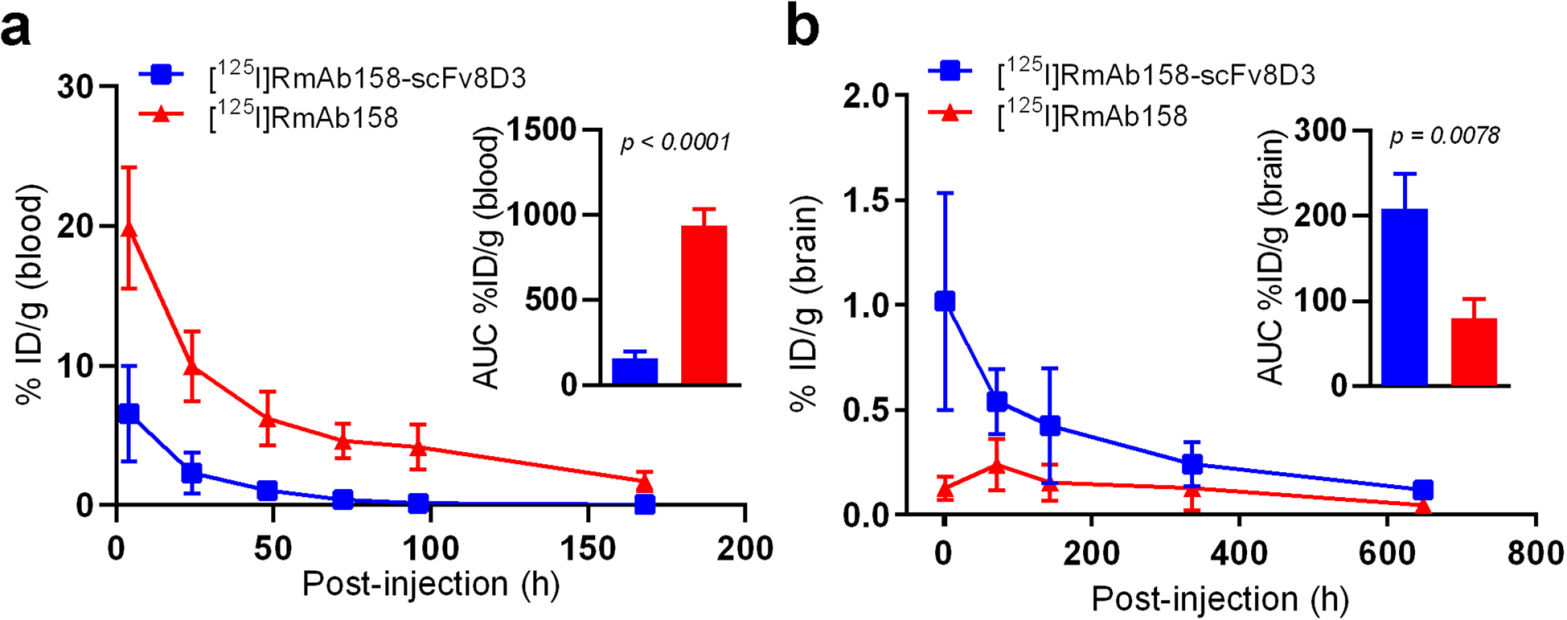
Ex vivo blood pharmacokinetics, expressed as percent of injected dose per gram blood, %ID/g (blood) (**a**), and brain retention, %ID/g (brain) (**b**) of [125I]RmAb158-scFv8D3 and [125I]RmAb158 over time. Inserts in (**a**) and (**b**) represent areas under the curve. Error bars represent SD and SEM (AUC figure)

Blood samples of mice injected with [^125^I]RmAb158-scFv8D3 or [^125^I]RmAb158 were obtained at 4 h, 24 h, 48 h, 72 h, 96 h, and 168 h post injection to investigate the concentration profiles of the two antibodies in blood, i.e. blood half-life and total drug exposure (AUC_blood_). [^125^I]RmAb158-scFv8D3 was more rapidly cleared from blood, compared with [^125^I]RmAb158 (Fig. 3a). The total drug exposure (AUC_blood_) of [^125^I]RmAb158 in blood from 4 h to 168 h post injection was 5.5-fold higher (*P* < 0.0001) than that of [^125^I]RmAb158-scFv8D3 (Fig. 3a).

Ex vivo measurement of brain tissue activity was performed to quantify the whole-brain retention of the antibodies. [^125^I]RmAb158-scFv8D3 (Fig. 3b, Table 2) displayed greater overall brain retention at all time points throughout the study, with high brain concentrations from as early as 2 h post injection (1.01%ID/g ± 0.52%ID/g), while the brain uptake of [^125^I]RmAb158 (Fig. 3b, Table 2) peaked on day 3 post injection (0.24%ID/g ± 0.12%ID/g) and then gradually decreased. The half-life in brain was comparable between [^125^I]RmAb158-scFv8D3 (7.5 days) and [^125^I]RmAb158 (8.6 days). Thus, despite faster blood clearance, the total brain exposure (AUC_brain_) of [^125^I]RmAb158-scFv8D3 was 2.59-fold higher than that of [^125^I]RmAb158 (*P* = 0.0078) (Fig. 3b).

**Table 2:** Ex vivo brain retention of [^125^I]RmAb158 and [^125^I]RmAb158-scFv8D3 (%ID/g)

Time post injection	[^125^I]RmAb158-scFv8D3	[^125^I]RmAb158
2 h	1.01 ± 0.52 (*n* = 3)	0.13 ± 0.05 (*n* = 3)
72 h	0.54 ± 0.16 (*n* = 8)	0.24 ± 0.12 (*n* = 7)
144 h	0.42 ± 0.28 (*n* = 8)	0.15 ± 0.09 (*n* = 5)
336 h	0.24 ± 0.11 (*n* = 5)	0.13 ± 0.10 (*n* = 5)
648 h	0.12 ± 0.04 (*n* = 7)	0.05 ± 0.01 (*n* = 5)

Data are presented as mean ± SD

### Immunostaining and ex vivo autoradiography

**Fig. 4:**
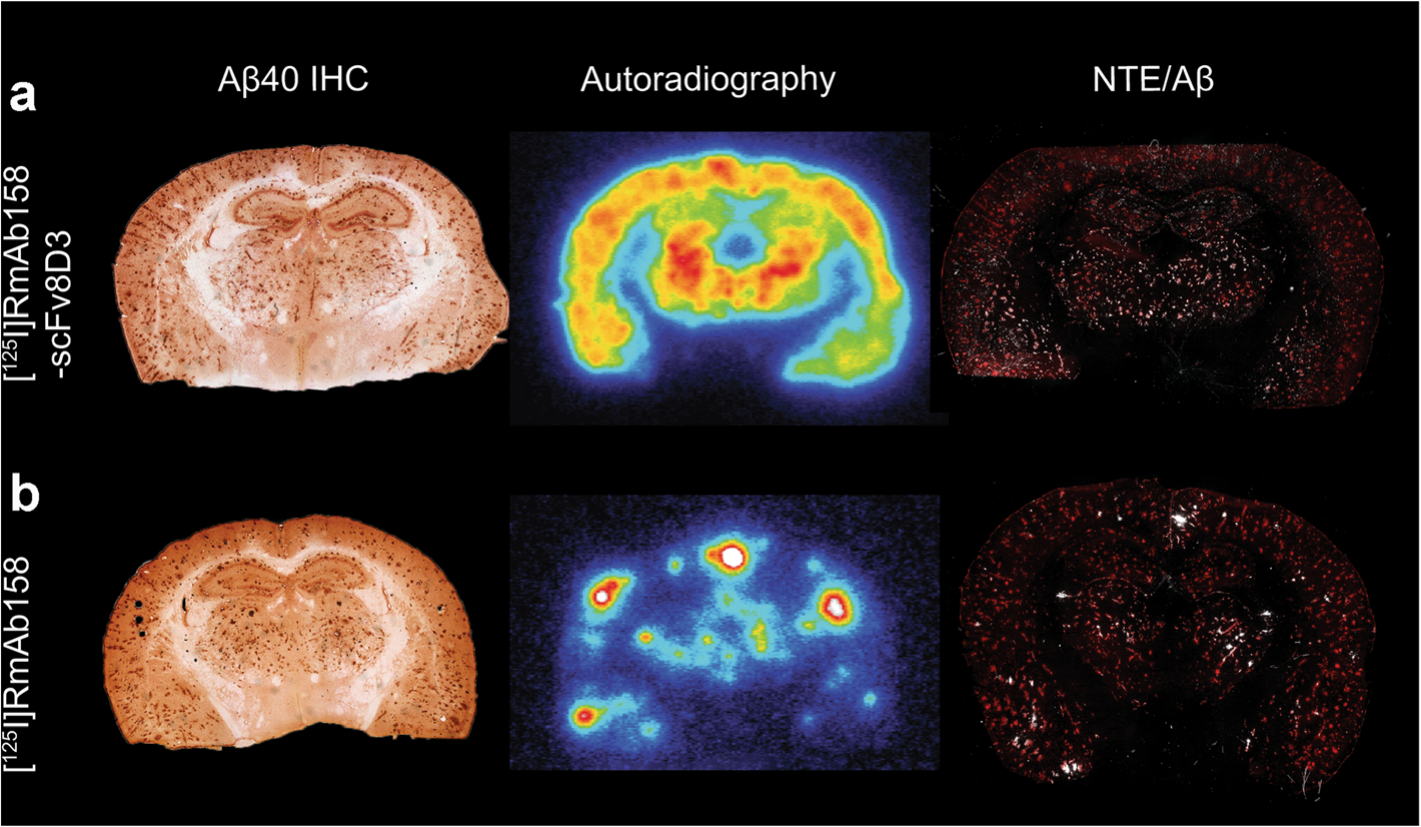
Brain distribution of [^125I^]RmAb158-scFv8D3 (**a**) and [^125I^]RmAb158 (**b**) in tg-ArcSwe mice, as revealed by Aβ40 immunohistochemical staining, ex vivo autoradiography 6 days post injection, and a merge of nuclear track emulsion (NTE) autoradiography (white puncta) in combination with Aβ immunofluorescence (red) 3 days post injection

Aβ immunostaining and ex vivo autoradiography were performed to further evaluate brain distribution of the antibodies in perfused mouse brain, devoid of the background blood activity. Immunohistochemistry results showed that Aβ40 was widespread throughout the cortex, hippocampus, and thalamus. [^125^I]RmAb158-scFv8D3 autoradiography, in concordance with SPECT results, showed a uniform radioactive distribution pattern in brain areas that harbored abundant Aβ pathology. NTE autoradiography combined with Aβ immunofluorescence was further performed to view the [^125^I]RmAb158-scFv8D3 signal at a higher resolution. This analysis confirmed co-occurrence of [^125^I]RmAb158-scFv8D3 with Aβ deposits in affected brain regions (Fig. 4a). Also, in line with SPECT, [^125^I]RmAb158 showed a different brain distribution pattern from [^125^I]RmAb158-scFv8D3, with high-intensity hotspots in areas with Aβ pathology, especially in cortex and thalamus, and in proximity to the lateral ventricles (Fig. 4b).

**Fig. 5:**
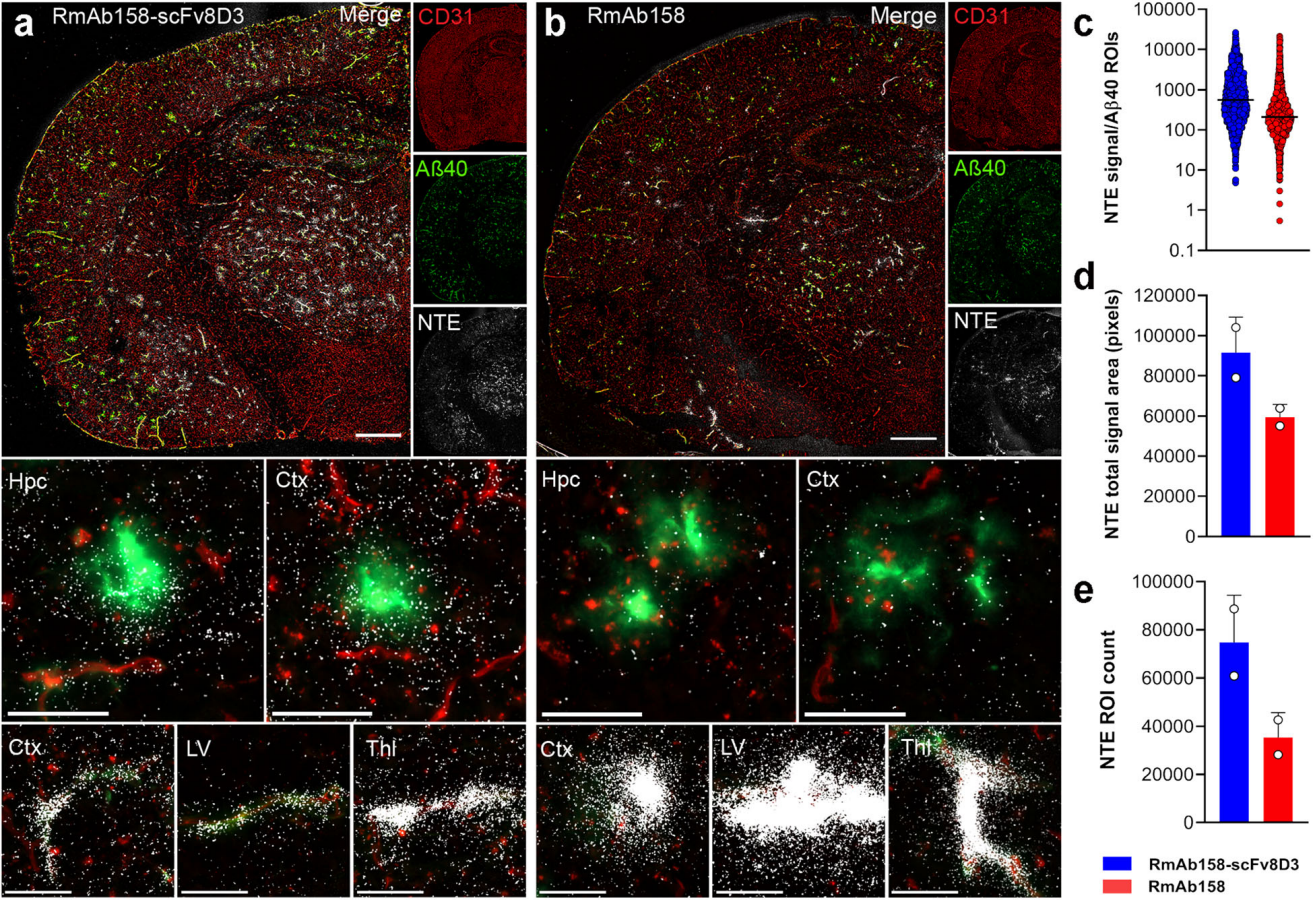
Nuclear track emulsion (NTE) autoradiography (white puncta) detection of [^125^I]RmAb158-scFv8D3 (**a**) and [^125^I]RmAb158 (**b**) in combination with Aβ40 (green) and endothelial cell marker CD31 (red) in tg-ArcSwe mice at 3 days post injection. Scale bars, 200 μm (brain hemispheres) and 50 μm (detailed images). **c** Quantification of [^125^I]RmAb158-scFv8D3 and [^125^I]RmAb158 co-localization with Aβ deposits. **d**, **e** Quantification of [^125^I]RmAb158-scFv8D3 and [^125^I]RmAb158 ROI areas (**d**) and numbers (**e**) in the brain parenchyma. The quantification data were from whole hemispheres of two mice

The brain distribution of [^125^I]RmAb158-scFv8D3 and [^125^I]RmAb158 in tg-ArcSwe brain was further assessed with NTE in combination with Aβ and CD31 immunofluorescence to visualize interactions of the antibodies with both Aβ and the brain vasculature (Fig. 5). At 3 days post injection, [^125^I]RmAb158-scFv8D3 exhibited extensive interaction with parenchymal Aβ around plaques in the cortex and hippocampus (Fig. 5a). As the tg-ArcSwe mouse model shows abundant vascular pathology, [^125^I]RmAb158-scFv8D3 also showed retention associated with Aβ deposits in cortical and thalamic vessels and to some extent in the ventricles (Fig. 5a). The [^125^I]RmAb158-scFv8D3 signal was evenly distributed throughout the brain, while [^125^I]RmAb158 showed distinct and abundant accumulation in a few, well defined hotspots in both cortical and thalamic vessels, co-localizing with Aβ deposits. [^125^I]RmAb158 was also abundant in proximity to the lateral ventricles, interacting with Aβ deposits (Fig. 5b). In line with the total antibody retention in brain (Fig. 3), [^125^I]RmAb158-scFv8D3 showed a greater degree of co-localization with Aβ in the brain compared with [^125^I]RmAb158 (Fig. 5c). Further, by subtracting CD31-positive regions from the images to limit the analysis to antibody-derived NTE signal in parenchymal (CD31-negative) regions, we confirmed a greater total distribution of [^125^I]RmAb158-scFv8D3 than [^125^I]RmAb158 in the parenchyma. This was evidenced by a larger total area covered (Fig. 5d) and a greater number of identified ROIs (Fig. 5e) for parenchymal antibody-derived NTE signal in [^125^I]RmAb158-scFv8D3- than in [^125^I]RmAb158-treated mice.

## Discussion

Previous imaging studies of RmAb158-scFv8D3 have focused on the first few days after antibody injection using ^124^I (half-life 4.2 days), which limits the possibility to image the antibody in the brain over longer periods of time. Here, we examined the brain retention and intrabrain distribution of the bispecific antibody RmAb158-scFv8D3, in comparison with the non-modified RmAb158 during 1 month, using the long-lived radionuclide ^125^I (half-life 59 days) for longitudinal SPECT imaging in tg-ArcSwe mice. Iodine is a non-residualizing radionuclide [28], which means that if it is separated from the protein, it will be secreted or taken up by the thyroid. It can therefore be assumed that the radioactive signal in brain tissue is derived from intact antibody residing in the brain.

In this study, in vivo SPECT imaging revealed different distribution patterns of [^125^I]RmAb158-scFv8D3 and [^125^I]RmAb158 in tg-ArcSwe mouse brain. As previously reported [18], [^125^I]RmAb158-scFv8D3 was rapidly transported across the brain vasculature endothelium, resulting in high brain concentrations at 2 h post injection, followed by elimination of unbound antibody. Approximately 50% of [^125^I]RmAb158-scFv8D3 that entered the brain was retained 3 days later. The high brain retention and fast blood clearance of [^125^I]RmAb158-scFv8D3 resulted in a clear pathology-associated SPECT signal in cortical, hippocampal and thalamic regions 3 days later. Interestingly, during the following month, there was a slow, gradual decrease in antibody retention in brain, and the uniform brain distribution of [^125^I]RmAb158-scFv8D3 was constant over time.

For [^125^I]RmAb158, although its brain concentration peaked at 3 days after injection, the prolonged blood residence caused a poor SPECT image quality due to a high blood-derived background at this time point. The [^125^I]RmAb158-injected tg-ArcSwe mice scanned at 14 and 27 days post-injection, when activity in blood had decreased and no longer interfered with the SPECT signal, displayed central, high-intensity accumulation of [^125^I]RmAb158 in the brain. In contrast to [^125^I]RmAb158-scFv8D3, [^125^I]RmAb158 initially accumulated at high-intensity hotspots in the brain, with only modest parenchymal distribution. While there was a gradual reduction and loss of cortical hotspots during the study period, central hotspots remained relatively stable. The low ventricular antibody retention and the absence of cortical hotspots in age-matched WT littermates suggested that Aβ pathology is a prerequisite for brain retention of antibodies. Indeed, NTE combined with Aβ immunostaining revealed that [^125^I]RmAb158 accumulations in cortical and thalamic hotspots reflected interactions with vascular Aβ deposits, i.e. CAA. Abundant [^125^I]RmAb158 interaction with Aβ was also observed in the ventricles, in line with previous observations [29]. Thus, the two antibodies seem to have a similar ability to interact with Aβ and be retained in an Aβ-bound state over a long period of time, with similar half-lives in the brain. This is supported by their high-avidity binding to Aβ protofibrils, with a very slow dissociation rate, as demonstrated by in vitro binding kinetic assays.

The difference in brain distribution between the antibodies is likely due to the differences in the transport routes by which they access the brain-associated Aβ deposits. RmAb158-scFv8D3 is transcytosed across the BBB, shuttled by TfR, which is highly expressed on endothelial cells of the brain capillaries [30], allowing the antibody to access the whole volume of the brain. However, this route does not apply to RmAb158. The pronounced retention of [^125^I]RmAb158 around the lateral ventricles could be due to its prolonged blood residence, in combination with the highly perfused [31] and permeable vasculature in the BCSFB, resulting in a high local influx of antibodies that could bind to ventricular Aβ. Furthermore, since [^125^I]RmAb158-scFv8D3 is transported throughout the whole brain vasculature, its local concentrations in both ventricles and areas with CAA are likely to be relatively lower than those of [^125^I]RmAb158. This is supported by the greater number of NTE ROIs and larger antibody-covered area in the brain parenchyma of transgenic mice injected with [^125^I]RmAb158-scFv8D3. Unmodified antibodies have been suggested to reach the brain primarily via CSF, along a perivascular route [32]. Thus, [^125^I]RmAb158 crossing the BCSFB could reach the perivascular space of large brain-penetrating vessels via CSF, favoring interactions with perivascular CAA.

ARIA-E observed in clinical immunotherapy trials is likely to be caused by antibody interactions with CAA. This phenomenon is particularly significant for aducanumab, which binds with high affinity to fibrillar Aβ deposits [33]. BAN2401 (humanized mAb158) favors interaction with soluble Aβ protofibrils and is generally well-tolerated in AD patients, with a low incidence of cerebral vascular edema: less than 10% in all dose-groups in the Phase IIb study [9]. Thus, the amount of CAA interactions observed in this study may not be translatable to the clinical situation, as we used a mouse model with very pronounced CAA pathology [34]. However, this indicates that the mechanism by which RmAb158-scFv8D3 is transported and distributed within the brain is likely to reduce the risk of potential therapy-induced BBB perturbations.

Once inside the brain, the fate of antibodies residing in the parenchyma is not fully understood, and multiple routes of elimination have been suggested, including neonatal Fc receptor-facilitated brain efflux [35] and degradation clearance due to Fcγ-activated microglial phagocytosis of the antigen-antibody complex [36]. RmAb158-scFv8D3 is effective in clearing soluble Aβ at a 10-fold lower dose than RmAb158 [23]. This process is fast (within 3 days) and may be mediated by elimination of soluble antibody-Aβ complexes by interstitial fluid flow or by Fc-mediated mechanisms. Here we show that approximately 50% of antibody entering the brain was eliminated during the first 3 days. The remaining half was retained, bound to Aβ deposits in the diffuse periphery of amyloid plaques. The brain concentration of [^125^I]RmAb158-scFv8D3 then gradually decreased throughout the study. Whether this decline is due to antibody dissociation from its target or to gradual clearance and degradation or elimination of antibody-Aβ complexes needs to be studied further. However, given the non-residualizing properties of ^125^I, RmAb158-scFv8D3 that interacts with extracellular Aβ followed by phagocytosis and degradation of antibody-Aβ complexes, e.g. by microglia, would result in a decline in radioactive signal.

From an imaging perspective, this study demonstrated that the brain retention of unmodified and bispecific antibodies labeled with the long-lived isotope ^125^I can be explored in vivo with SPECT. Traditional brain imaging ligands are usually small molecular compounds, radiolabeled with the relatively short-lived carbon-11 (^11^C) or fluorine-18 (^18^F), with nanomolar affinities. These radioligands have a biological and radioactive half-life in the range of minutes to hours, whereas the ^125^I-labelled antibodies used in this study have a biological and radioactive half-life in the range of days to weeks, which implies the benefit of this imaging technique for long-term studies of brain uptake and retention of antibodies. With the help of long-lived radionuclide in combination with SPECT scanning, the complete dynamics of brain distribution of the two antibodies could be followed up in vivo over a period of 1 month*.* Combined with the picomolar affinity of antibodies to their target, this experimental setup provides a possibility to investigate and monitor neurobiological processes in vivo over a long period of time. Further, although RmAb158 was found to be co-localized with Aβ in relatively high concentrations, its distribution was not representative of the overall distribution of Aβ pathology in the brain. Therefore, a system that can actively transport antibody into the brain is essential for making use of an antibody as an imaging agent for intra-parenchymal targets.

## Conclusions

This study demonstrates how long-term SPECT imaging in combination with ex vivo autoradiographic techniques and immunostaining can be used to study long-term distribution of antibodies in the brain. The results suggest that RmAb158-scFv8D3 and RmAb158 reach different parts of the brain by different routes, which has mechanistic implications for their use as therapeutics. Further, the increased brain exposure and enhanced spatial distribution of the bispecific, brain-penetrating RmAb158-scFv8D3 demonstrate that the antibody-based imaging of the central nervous system requires active transport of antibody into the brain. These findings also suggest that such bispecific antibodies could be used in a theranostic setting, where the same antibody is used for both imaging and therapy.

## Data Availability

The datasets used and/or analysed during the current study are available from the corresponding author on reasonable request.

## Abbreviations

Aβ: Amyloid-β
AβPP: Aβ protein precursor
AD: Alzheimer’s disease
ARIA-E: Amyloid related imaging abnormalities – edema
BBB: Blood-brain barrier
BCSFB: Blood-cerebrospinal fluid barrier
CAA: Cerebral amyloid angiopathy
scFv: Single-chain variable fragment
SPECT: Single photon emission computed tomography
TfR: Transferrin receptor
tg-ArcSwe: AβPP transgenic mice with the Arctic and Swedish AβPP mutations
